# A conceptual framework for designing Ambient assisted living services for individuals with disabilities in Uganda and South Africa

**DOI:** 10.4102/ajod.v8i0.477

**Published:** 2019-08-26

**Authors:** Michael Kyazze, Janet Wesson, Kevin Naudé

**Affiliations:** 1 Department of Computing Sciences, Nelson Mandela University, Port Elizabeth, South Africa

**Keywords:** disability, requirements identification, ambient assisted living, smart home, quadriplegia

## Abstract

**Background:**

Individuals with disabilities experience difficulty in using various everyday technologies such as computers and smartphones.

**Objectives:**

To propose a conceptual framework that will lead to the development of practical and user friendly assistive technology.

**Method:**

A literature review of challenges faced by individuals with physical disabilities was carried out. Interviews with adults with physical disabilities in Kampala, Uganda, and Port Elizabeth, South Africa, identified three main challenges with regard to using technology: using a mobile phone, controlling an electronic environment and using a computer.

**Results:**

The challenges identified can be solved by taking into consideration the needs of individuals with disabilities. However, the design of new technologies and interaction techniques, such as natural hand gestures and voice, as input mechanisms has able-bodied individuals in mind. Individuals with disabilities are considered as an afterthought. The main reason for this is that individuals with a disability are a minority and hence it may not make economic sense for technology innovators to cater for their unique needs. A lack of practical guidelines on how to design for individuals with disabilities is another reason why designing for individuals with disabilities is often an afterthought.

**Conclusion:**

This article proposes a conceptual framework that can be used by researchers and technology designers in order to design products that could cater for the unique needs of individuals with disabilities. The article also emphasises the importance of exploring alternative interaction techniques, as they could enable individuals with disabilities to fully utilise technologies such as smart phones, computers and smart home electronics.

## Introduction

There are over 650 million estimated people with disabilities worldwide (World Health Organization [WHO] & World Bank 2011). South Africa has an estimated disability prevalence rate of 7.5%, excluding psychosocial and cognitive disabilities. This implies that over 2.8 million South Africans face difficulties related to hearing, vision, communication, walking, climbing stairs, remembering and self-care (Statistics South Africa 2011). Nine per cent of the Ugandan population are estimated to have some form of disability (Uganda Bureau of Statistics [UBOS] and International classification of functioning [ICF] 2011), which impacts their daily lives.

The World Health Organization (WHO) and World Bank state that the definition of disability is complex, dynamic, multidimensional and contested (WHO & World Bank 2011). Disability is defined as an umbrella term for impairments, activity limitations and participation restrictions, which limit the interaction between an individual and the individual’s contextual factors (environmental and personal factors) (Ustun 2001). Physical disability refers to loss or lack of limbs and damage to muscles, nerves, skin or bones that lead to difficulties moving about and in performing activities of daily living (such as dressing, eating, cleaning, etc.) (Dpsa 2001). The nature of physical disabilities includes *skeletal* disability, such as joint movement limitations, small limbs, missing limbs or abnormal trunk size, and *neuromuscular* disability, which is caused by ailments that affect muscular control in part or most of the body.

**FIGURE 1:**
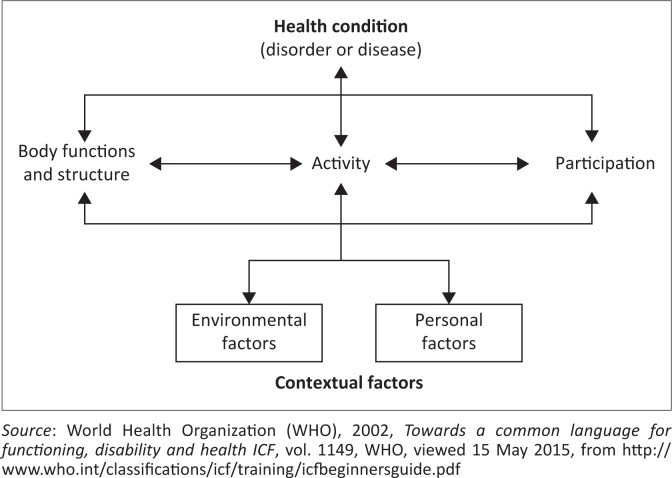
The International classification of functioning, disability and health framework.

Personal care and mobility challenges are experienced every day by individuals with disabilities, while challenges in using technology may be experienced with different frequencies. Physical disabilities may be caused by a number of factors, such as accidents, disease, congenital disorder and spinal cord injury. An alternative for identifying challenges from the literature is by using the International Classification of Functioning, Disability and Health (ICF). The ICF is a framework for describing and organising information on functioning and disability. The ICF helps to describe what individuals with disabilities can do in a standard environment (their level of capacity), as well as what they actually do in their usual environment (their level of performance) (WHO 2002). Figure 1 illustrates the ICF. Environmental factors represent a physical, social and attitudinal environment in which people live and conduct their lives.

The ICF has been used as a basis for the design of a framework for physiotherapy management (Harvey 2007). The ICF may be used to gain a better understanding of how a particular disability or health condition affects individuals, for example considering quadriplegia (Langtree 2010). An associated impairment is limited hand use. Limited hand use directly impacts the ability to perform activities such as personal care and mobility. This in turn has implications for participation, such as working, studying and engaging in family life. Impairments, activity limitations and participation restrictions are all affected by environmental and personal factors, such as the availability of support aids, support from family and source of livelihood for the individual with a disability. The above discussion shows that ICF can help in the identification of challenges faced by individuals with disabilities.

One way of overcoming the challenges faced by individuals with disabilities is by employing personal assistants. However, the cost of employing a personal assistant on a 24 h basis is beyond the financial reach of most people. South Africa provides a care dependency grant to primary care givers (personal assistants) of children with disabilities, who require permanent care but do not reside in state-run institutions. In 2011, nearly 111 000 children received the grant, which was R1200 per month ($86) (DWCPD and UNICEF 2012; Western Cape Government 2012). Unlike South Africa, which provides a monthly grant to individuals with disability, Uganda does not currently provide financial assistance to cover costs associated with daily living (Abimanyi-Ochom & Mannan 2014). However, Uganda piloted a social empowerment programme between 2011 and 2015 that provided 25 000 Uganda shillings per month ($6.50) to vulnerable members of society, such as individuals with disabilities and the elderly (Brook, Jones & Merttens 2012). Such amounts may not be enough to cater for basic needs, such as food and shelter.

Assisted living may be defined as a system of housing and care that is designed for the elderly, or individuals with disabilities, and offers various levels and combinations of services, care and privacy (Carpenter et al. 2006). Assisted living may be enhanced by various technologies, such as Ambient Intelligence. Ambient Intelligence may be defined as the presence of a digital environment that is sensitive, adaptive and responsive to the presence of people (Cook, Augusto & Jakkula 2009). Ambient assisted living (AAL) is the use of Ambient Intelligence to extend the time which the elderly and individuals with disabilities can live independently in their preferred environment (AAL-Europe 2013b; Memon et al. 2014). Some of the application areas of Ambient Intelligence include education, emergency services, transportation, hospitals, health monitoring and assistance, work places and smart homes (Cook et al. 2009). One of the application areas of AAL is a smart home environment. A smart home environment is one that integrates diverse, context-aware, automated technology and services with the aim of unobtrusively enhancing the lives of its inhabitants (Alam, Reaz & Ali 2012).

The application of AAL technology to provide assistance to individuals with diminished independence is a growing area of research. In order to guide solution developers in creating smart home environments, research projects were undertaken to better understand the needs of the intended users. The UniversAAL project was one such project, which was a European research project (2010–2014), with the goal of building consensus among the AAL community, and consolidating their efforts to produce technically feasible and economically affordable standardised AAL systems (Tazari, Furfari & Valero 2012). The UniversAAL project provides a suitable technical context where solutions may be developed for individuals with disabilities. The UniversAAL platform, which consists of runtime support, development support and community support, and a reference architecture were the main deliverables of the project (UniversAAL 2010).

The main components of the UniversAAL project are the following:

- AAL services: these are software artefacts that address a specific need in an AAL space (e.g. home, car and hospital).
- Network artefacts: these are gadgets that implement or contribute to the implementation of AAL services (e.g. sensors).
- AAL space: an environment that provides AAL services with the help of embedded networked artefacts (e.g. home, car and hospital).
- AAL Reference Architecture: this identifies the basic building blocks necessary for constructing an AAL space.
- AAL platforms: software that implements the AAL Reference Architecture in order to provide for resource sharing and allow users to experience an integrated world based on natural communication.

**FIGURE 2:**
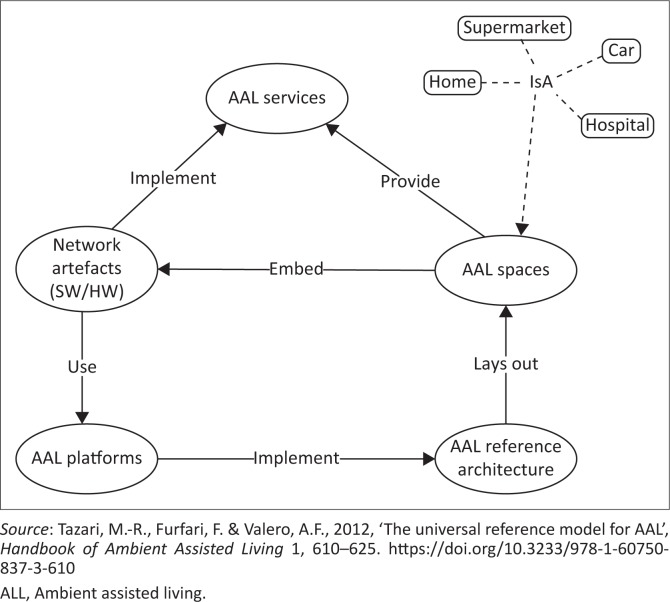
The UniversAAL Reference Architecture.

The AAL Reference Architecture identifies the basic building blocks necessary for constructing an AAL space, such as home, supermarkets or hospitals (see Figure 2). The AAL space provides AAL services with the help of embedded networked artefacts. The cooperation between networked artefacts distributed in an AAL space is facilitated by an AAL platform. The reference architecture can be used as a foundation for designing AAL solutions for disabled individuals. Ambient assisted living may enable disabled individuals to be less dependent on personal assistants. Ambient assisted living innovations can be made up of hardware and software components. The term ‘AAL services’, as used in this article, specifically refers to AAL software. The involvement of users in solution design is essential in ensuring the success of that solution. The next section discusses the interview studies carried out with individuals with disabilities and their personal assistants in Uganda and South Africa.

## Interview study

The study was interpretive in its philosophical approach and employed a qualitative methodology involving interviews. The purpose of the interviews was to gain a better understanding of the challenges faced by individuals with disabilities and to contextualise the findings from the literature.

### Participants

A purposive sampling method was employed in this study. This process involves selecting cases that will best enable the research question to be answered or objectives to be met. That is, the sample size is sufficient when additional interviews do not result in identification of new concepts, an end point called data saturation. To determine when data saturation occurs, analysis ideally occurs concurrently with data collection in an iterative cycle. This allows the researcher to document the emergence of new themes and also to identify perspectives that may otherwise be overlooked. Eighteen individuals with disabilities and 12 personal assistants were interviewed. The assistants were all close relatives. Participation in the interviews was restricted to individuals with physical disabilities. The interviews were conducted in two different African urban cities, namely Kampala, Uganda, and Port Elizabeth, South Africa. The Ugandan participants were recruited with the help of a community social worker, while the South African participants were recruited through a disability support centre at the Nelson Mandela University.

A scientific classification of physical disabilities is essential for studying the challenges that individuals with disabilities experience (Table 1). The classification is used to describe the participants’ level of disability.

**TABLE 1:** Spinal cord injuries.

Level of injury	Possible impairment
C2–C3	Usually fatal as a result of inability to breathe
C4	Quadriplegia and breathing difficulty
C5	Quadriplegia with some shoulder and elbow function
C6	Quadriplegia with shoulder, elbow and some wrist function
C7	Quadriplegia with shoulder, elbow, wrist and some hand function
C8	Quadriplegia with normal arm function; hand weakness
T1–T6	Paraplegia with loss of function below mid-chest; full control of arms
T6–T12	Paraplegia with loss of function below the waist; good control of torso
L1–L5	Paraplegia with varying degrees of muscle involvement in the legs

*Source*: University of Rochester, n.d., *Spinal Cord Injury,* viewed 29 March 2016, from https://www.urmc.rochester.edu/neurosurgery/for-patients/conditions/spinal-cord-injury.aspx

C2–C8, cervical level 2–8; T1–T12, thoracic level 1–12; L1–L5, lumbar level 1–5.

The spinal cord injuries described in Table 1 require different levels of care and support aids. Individuals with disabilities who have cervical (C) injuries between C2 and C4 require the most care, while individuals who have thoracic (T) or lumbar (L) injuries require less care. Individuals with C-level injuries may benefit more from assistive technology solutions as compared to individuals with T and L injuries, because of the extent of their need for assistance.

### Data collection and analysis

Four interview questions for individuals with disabilities were formulated from the findings of a literature review study on challenges faced by individuals with disabilities in their day-to-day lives. The questions were supported with probing questions in order to obtain more information from the participants in situations where they gave brief responses to the main questions. An audio recorder was used to record the interviews. The interview questions were as follows:

- What challenges do you deal with on a daily basis and how do you cope with them?
- What support aids do you normally use and how do you use them?
- What are some of the limitations of the support aids?
- How would you address some of the limitations of these support aids?

The interview questions were limited to activities carried out in living room and kitchen environments. This was done so that participants did not have to share experiences that might have made them feel uncomfortable. The interview questions for personal assistants were the following:

- What kind of assistance do you offer to disabled individuals?
- How often do you provide assistance remotely and what technology do you use?
- What kind of assistance do you offer remotely?
- What other ways do you think technology can help you to provide better assistance?

The interviews were carried out over a 2-month period in 2016. The interviews were recorded and transcribed, and the transcriptions were made verbatim. Computer-assisted qualitative data analysis using Atlas.ti version 8 was employed to organise the data and facilitate thematic coding and identification of themes.

## Results

A summary of the themes identified from the interview studies is presented here:

- Personal care: this is concerned with how individuals take care of themselves, for example some use personal assistants and relatives.
- Mobility: this is related to how individuals move around from one place to another, for example by using wheel chairs.
- Social and economic well-being: this is concerned with how individuals provide for themselves, for example through government disability grants.
- Communication: this is related to how individuals are able to communicate with others, for example by using sign language.

Personal care and mobility stood out in both the literature study and interviews as the main challenges facing individuals with disabilities. A detailed discussion of the interview findings is provided next.

### Ugandan interview study findings

Table 2 provides a summary of the disabilities of the participants. Nine individuals with disabilities and nine personal assistants were interviewed; four of the participants with a physical disability were women, while five were men. All the participants with physical disabilities lived in private homes and were cared for by their relatives. Five of the participants had spinal cord-related injuries.

**TABLE 2:** Characteristics of participants in Uganda.

Participant	Disability
U-P1	No lower limbs because of accident (female)
U-P2	L1 spinal cord injury (female)
U-P3	T6 spinal cord injury (male)
U-P4	No lower limbs (male). He was born without lower limbs.
U-P5	No lower limbs (female). She was born lame.
U-P6	Immobile limbs (male)
U-P7	L1 spinal cord injury (female)
U-P8	L1 spinal cord injury (male)
U-P9	C6 spinal cord injury (male)

C, cervical level; T, thoracic level; L, lumbar level.

The findings from the Ugandan interview studies are described below and grouped according to themes identified.

Personal care: U-P2 has difficulty in bending to do things like washing clothes and picking up items from the ground. She does not have any support aids. She said that her life could be made easier if she could get a washing machine and a dishwasher. She currently relies on her teenage daughter for assistance.

Mobility: U-P1 is unable to climb stairs without assistance; she relies on assistance to get items that she cannot reach, for example cooking items that are on shelves. She wants a self-drive wheelchair, which she can use without assistance. U-P5 crawls since she has no wheel chair. She relies on her nephew for assistance with activities such as cooking and cleaning. U-P6 experiences difficulty in walking without assistance; he currently uses a crude wooden support. He wants a modern walking stick. UP-7 cannot walk; she does not want a wheelchair or a walking stick because she was not born lame, but became paralysed because of sickness. UP-8 uses a walking stick; his wife helps him with most of the household activities so that he does not have to move around unnecessarily. UP-9 has no wheelchair, so he crawls to move about. He would like a battery-powered wheelchair so that he can move without assistance.

Social and economic well-being: U-P3 feels emotional pain and abandoned by his family because he needs continuous support. He is unable to find a meaningful employment. He was a motorcycle taxi operator before he suffered an accident. U-P4 experiences discrimination by the public; he needs a tricycle which he can use by himself without assistance. U-P7 experiences discrimination from some members of the public.

The participants experienced difficulty in moving from one place to another, and emotional pain and difficulty in carrying out personal care activities. Personal care and mobility were two of the challenges identified from the literature. The nine personal assistants reported that the primary assistance they provide is with mobility and personal care-related challenges. The interviewer discussed AAL with the participants. The participants agreed that AAL services, such as turning on or turning off lights without assistance and controlling TV sets without assistance, may be helpful to them. They emphasised that their most important needs are support aids, such as wheelchairs, walking sticks, prosthetics and battery-powered wheelchairs. These would help them to be less reliant on assistance from relatives. They attributed the lack of these essential aids to poverty.

### South African interview study

Nine disabled individuals and three personal assistants were interviewed in Port Elizabeth, South Africa. Seven of the disabled individuals were men, while two were women. Six of the participants were residents of a home for the disabled, while three were university students. Six of the participants had spinal cord-related injuries. Table 3 provides a summary of the participants’ disabilities.

**TABLE 3:** Characteristics of South African participant disability.

Participant	Disability
S-P1	T6 spinal cord injury (male)
S-P2	C5 spinal cord injury (male)
S-P3	C6 spinal cord injury (male)
S-P4	C7 spinal cord injury (female)
S-P5	C6 spinal cord injury (male)
S-P6	C7 spinal cord injury (male)
S-P7	Short limbs because of polio (male)
S-P8	Cerebral palsy (male)
S-P9	Short limbs because of polio (female)

The findings from the South African interview studies are described below and grouped according to themes identified.

Personal care: S-P9 needs assistance to get items, such as plates from shelves. She has a wheelchair but cannot self-propel.

Mobility: S-P1’s lower body is immobile (waist downwards). He uses a wheel chair to move around. He does not want a battery-powered wheelchair because he says that it will make him lazy. He drives himself to and from school using a specially modified car. He needs assistance in cooking; he says that while he is able to use his arms, they usually become tired because he uses them for everything. S-P5 is unable to use his left hand. He currently uses a manual wheelchair; he would like an electric wheelchair but cannot afford one. He needs assistance to get out of the wheelchair. He is also unable to cook for himself. S-P7 is able to fully use his hands; however, he is confined to a wheelchair because of polio. He uses a picker to pick up items from the ground; however, he needs assistance to get items, such as books from shelves.

Communication: S-P1 said that he would find it beneficial if he could use voice commands and gestures to interact with electronic devices, such as a TV. S-P2 lives in a disability care home, and he is unable to speak clearly. He uses a head-mounted device to interact with a computer. He relies on caregivers to assist him with putting on and removing the head gear. S-P3 is unable to use his phone fully as he has limited control of his hands and cannot press the buttons. He is able to interact with a computer using a mouth stick. He needs to be fed by a caregiver. S-P3 complained that he normally uses loud speakers on his phone because he cannot hold the phone to his ears. S-P4 has difficulty in using the phone and also experiences difficulty when using a computer. S-P6 is unable to use his hands fully. He says that voice commands and gestures would be very useful. He occasionally uses a tablet computer. He says that tablets are much easier to use because they use gestures, but it takes time and is frustrating. S-P8 is unable to answer his mobile phone without assistance, as he cannot hold the phone. He uses special magnifying software on his computer. He says that a voice-operated system would be helpful for him. He uses a custom battery-powered wheelchair, which has a working area (table), where he places his laptop and study books. He needs assistance to get his laptop to and from his work area. He uses a special program, called the GRID, to enable him to use a computer.

### Recommendations

It was noted that individuals with the same disability experienced similar challenges, but required different levels and types of support. Some of the activities mentioned by the participants can be addressed using assistive technology:

- Answering a phone call without assistance (S-P2 and S-P8).
- Having a phone conversation without using a loud speaker (S-P3).
- Using a mobile phone or tablet computer without the need to use swipe gestures (S-P6).
- Using a mouth stick to interact with a computer (S-P3).
- Improving the efficacy of a head-mounted pointer to control the keyboard of a computer (S-P2).
- Enabling individuals to communicate with others, physically and electronically (S-P2).
- Turning lights on or off, controlling a TV and controlling a radio (S-P2 to S-P6).

S-P6 said that ‘head gestures and voice commands will help a wide variety of disabled individuals to do small things without assistance’. The participants discussed how their support aids help them to be less dependent on others. The Ugandan study discovered that the main challenge that disabled individuals experience is a low quality of life, as they are unable to afford support aids such as wheelchairs and walking sticks. They said that technology support would be good, but would not meet their immediate need for basic support aids. In contrast, the South African participants, having essential support aids, were more positive that AAL could improve their lives.

Depending on the body functioning capabilities of the target users, innovative interactive techniques may need to be developed. For example, a large part of human body language communication is the use of head gestures and facial expressions, and most cultures use subtle head movements to convey meaning (Paggio & Navarretta 2011). Some disabled individuals may benefit from such an interaction mechanism (Davis & Vaks 2001; Wei et al. 2013).

The challenges identified from the interview studies that could be addressed using assistive technology are summarised as software design requirements and listed in Table 4. Individuals with physical disabilities may require different interaction techniques depending on their body limitations. One of the observations from the interaction with individuals with disabilities is the various ways in which they may interact with a similar object of interest. For example, an individual with limited hand use may use his or her feet to turn on a radio, while another individual with limited hand and lower torso use may use his or her mouth to turn a radio on.

**TABLE 4:** Software design requirements.

Number	Challenges in using technology	Requirements	Possible interaction techniques
1	Answering a phone call without assistance	Using a mobile phone	Voice, head shake and nod, facial expression
2	Having a phone conversation without using a loud speaker	Using a mobile phone	Voice, head shake and nod, facial expression
3	Using a mobile phone or tablet computer without the need to use swipe gestures	Using a computing device, using a mobile phone	Voice, head shake and nod, facial expression
4	Using a head-mounted pointer to control a computer’s keyboard	Using a computing device	Voice, head shake and nod, facial expression
5	Facilitating communication	Using a mobile phone	Voice, head shake and nod, facial expression
6	Turning on or off lights	Controlling an electronic environment	Voice, head shake and nod, facial expression
7	Controlling a television and radio	Controlling an electronic environment	Voice, head shake and nod, facial expression

Interaction is a way of framing the relationship between people and objects designed for them, and thus a way of framing the activity of design (Dubberly, Pangaro & Haque 2009). While able-bodied individuals can freely use their hands to interact with technology using keyboards, computer mouse and swipe gestures, among others, individuals with disabilities may not be able to use these interaction techniques. Innovative interaction techniques may need to be considered. The design process of AAL services should explore different interaction techniques that may be used by the target users and choose interaction techniques that work best.

Saleh and Berns (2015) proposed an interaction model for facilitating communication between a humanoid robot and a human, both verbally and non-verbally. Head gestures were used as feedback for the robot to adapt the interaction scenario. This is an example of using the head shake and nod as an interaction technique. Facial features such as the nose and lips can be used as interaction techniques by individuals with disabilities (Gips & Margrit 2007). The next section proposes a conceptual framework that may be used when deciding which interaction techniques are suitable for a specific group of individuals with disabilities.

## Proposed conceptual framework

A framework may be defined as a system of concepts, assumptions, expectations, beliefs and theories that support and inform a research study (Maxwell 2013). In multidisciplinary research, multiple bodies of knowledge belonging to different disciplines are explored. A conceptual framework can help in the better understanding of the phenomenon being investigated. The main features of a conceptual framework are (Jabareen 2009) the following:

- It is a construct in which each concept plays an integral role.
- It provides an interpretive approach to social reality.
- Rather than offering a theoretical explanation, as do quantitative models, conceptual frameworks provide an understanding.
- A conceptual framework does not provide knowledge of hard facts but rather ‘soft interpretation of intentions’.
- Conceptual frameworks are indeterminist in nature and therefore do not enable us to predict an outcome.
- Conceptual frameworks can be developed and constructed through a process of qualitative analysis.
- The sources of data consist of many discipline-oriented theories that become the empirical data of the conceptual framework analysis.

**FIGURE 3:**
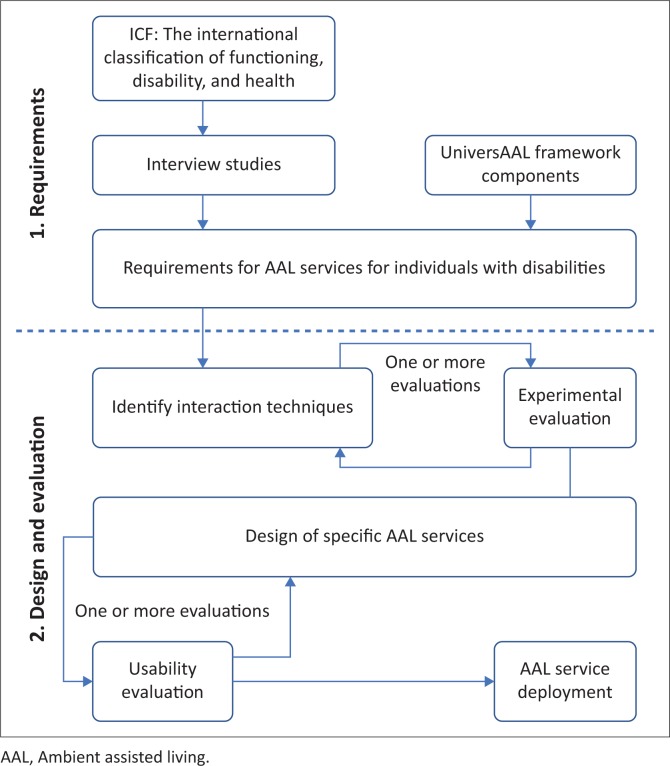
A framework for designing AAL services for individuals with disabilities.

Figure 3 illustrates the proposed framework for designing AAL services for individuals with disabilities. The framework is divided into two sections: requirements and design. Requirements are concerned with identifying and contextualising software requirements, while design is concerned with delivering usable software for the target users. The various components of the framework are presented below:

- ICF: the ICF helps to clearly define the characteristics of the target participants. The characteristics include body functioning and structure. This information is then used to narrow down the scope of a literature study on disability challenges.
- Interview studies: challenges identified from the literature may lack contextual information about a researcher’s intended target audience. The challenges can be used to design open-ended and non-leading questions. A number of probes can also be created from the challenges identified from the literature. The probes are necessary in situations when participants give short answers to questions. Probes can help reveal more information.
- UniversAAL framework components: the analysis of challenges from the literature and interview studies informs the decision on which UniversAAL components should form part of the technological requirements. The UniversAAL components are not exclusive; rather they provide a starting point to think about a solution.
- Requirements: a set of tasks that the intended AAL services aim to support.
- Identify interaction techniques: interaction is the way through which individuals are able to use the AAL services. The requirements and body limitations of the disability target group inform the initial set of proposed interaction techniques.
- Experimental evaluation: it helps identify practical and usable interaction techniques and also excludes unpractical ones. Low-level prototypes or basic software artefacts that use the proposed interaction techniques are designed and implemented. The intended users (individuals with disabilities) are involved in the evaluation of the interaction techniques in order to identify the most suitable one for a given task. One or more evaluations may be carried out.
- Design and implementation of specific AAL services: the selected interaction techniques are used to design and implement the AAL requirements.
- Usability evaluation: a usability evaluation study is carried out with the target set of participants. The feedback received may be used to improve the artefact. More evaluation studies can be carried out to the satisfaction of the solution developers.
- AAL service release: the developed AAL service may be released to the public or for private use.

While the descriptions of the components may suffice for some technical users, a guideline on how to use the framework can make it easier to apply. A guideline is presented below and is grouped into two sections: requirements, and design and evaluation.

### Requirements

The upper section of the framework is called ‘Requirements’. This is because it is concerned with gaining an in-depth understanding of the problem that is being addressed and how the target group of users currently experience and cope with the problem (phenomenon under investigation). Identifying requirements involves the following steps:

- Describing persons with a disability in terms of the ICF framework: this helps a researcher or solution developer to have a uniform and scientific way of describing the intended users of an artefact.
- Literature review: A description of the intended users is used to narrow down the literature on the types of challenges faced by a group of individuals with disabilities.
- Interview protocol: Using the knowledge obtained from the literature study, interview questions are formulated. The interview questions should be designed in such a way that a researcher can gain a better understanding of the phenomenon under investigation.
- Interviewing target users: The protocol is used to interview persons with disabilities, and an analysis of the interview results is used to come up with system requirements of an artefact.
- Technical requirements: The UniversAAL framework is used to identify key AAL components that can address requirements identified from the interview studies.

### Design and evaluation

The lower section of the framework is called ‘Design’. This is because it is concerned with designing an artefact that fulfils the identified requirements. Design involves the following steps:

- Identification of interaction mechanisms: Low-level prototypes can be used to understand the practicality of interaction mechanisms that are envisioned by a researcher. Less practical interaction mechanisms can be eliminated during this stage.
- Experimental evaluation of interaction mechanisms: A prototype of the various interaction mechanisms can be developed and evaluated with a small set of intended users. This can help a researcher to understand how the interaction mechanisms are likely to be received by the users. The researcher can make changes as needed.
- Development of artefact and usability evaluation: Once the interaction mechanisms are confirmed, all the features of the artefact can be developed. A usability evaluation is then carried out. The feedback from the evaluation can determine if improvements to the design are necessary; if not, the artefact can then be used by the target users.

## Discussion

Disability support aids, such as wheelchairs and walking sticks, have gone a long way in enabling individuals with physical disabilities to partake in activities of daily living, such as mobility and personal care. One of the findings from interview studies carried out with individuals with disabilities and their assistants is the need to have technology innovations more accessible to individuals with physical disabilities.

The emergence of smart home technologies and its associated interaction mechanisms, such as voice and facial gesture recognition, and context awareness will usher in a new era where accessibility requirements can be met by the nature of the interaction mechanisms. Some individuals with a disability may be able to use voice commands for interaction with a smart home environment. For example, AAL technology innovations are helping elderly individuals to live independently in their preferred home environments instead of moving into care homes (AAL-Europe 2013a). A number of AAL innovations use innovative interaction techniques, such as reading an individual’s head movements and voice commands, in order to execute commands, such as turning a TV on or off. While some of these AAL innovations can also benefit individuals with disabilities, they are designed for able-bodied individuals and do not take into consideration the physical body limitations of people with disabilities. The framework proposed in this article aims to address this gap by proposing a design framework that may help technology innovators to better understand the needs of individuals with disabilities.

Socio-economic factors of persons with disabilities affect their views on AAL innovations. In the case of Uganda and South Africa, it is important to design AAL innovations that are affordable by the target users. The proposed framework emphasises the view that individuals with a similar disability may require different ways to interact with the same technology. Hence, it is important to have a narrow set of intended target users who can fully benefit from the innovation, rather than a more general set of users who find limited benefits from the innovation. There is a need to have more accessible technology to cater for the needs of individuals with physical disabilities (Dobransky & Hargittai 2017).

## Conclusion

This article explored the challenges faced by individuals with disabilities, and specifically challenges that may be addressed by using technology. The challenges identified are similar to those found in the existing literature. However, interviewing the participants provided insights into how the socio-economic status of individuals affects their experiencing challenges in daily life. The results of the interviews were used to develop a framework for designing AAL services. The framework may assist other researchers and solution developers through the process of requirements gathering up to deployment of the artefact. Future studies can make use of the proposed framework as a starting point when designing technology solutions for individuals with disabilities.
